# High quality draft genome sequence of *Leucobacter chironomi* strain MM2LB^T^ (DSM 19883^T^) isolated from a *Chironomus* sp. egg mass

**DOI:** 10.1186/s40793-015-0003-3

**Published:** 2015-05-08

**Authors:** Sivan Laviad, Alla Lapidus, Alex Copeland, TBK Reddy, Marcel Huntemann, Amrita Pati, Natalia N Ivanova, Victor M Markowitz, Rüdiger Pukall, Hans-Peter Klenk, Tanja Woyke, Nikos C Kyrpides, Malka Halpern

**Affiliations:** 1 Dept. of Evolutionary and Environmental Biology, Faculty of Natural Sciences, University of Haifa, Haifa, Israel; 2 Theodosius Dobzhansky Center for Genome Bioinformatics, St. Petersburg State University, St. Petersburg, Russia; 3 Algorithmic Biology Lab. St. Petersburg Academic University, St. Petersburg, Russia; 4 Dept. of Energy Joint Genome Institute, Genome Biology Program, Walnut Creek, CA, USA; 5 Biological Data Management and Technology Center, Lawrence Berkeley National Laboratory, Berkeley, California, USA; 6 Leibniz Institute DSMZ – German Collection of Microorganisms and Cell Cultures, Braunschweig, Germany; 7 Dept. of Biological Sciences, Faculty of Science, King Abdulaziz University, Jeddah, Saudi Arabia; 8 Dept. of Biology and Environment, Faculty of Natural Sciences, University of Haifa, Oranim, Kiryat Tivon, Israel

**Keywords:** Leucobacter chironomi, Microbacteriaceae, Chironomid, Chironomus, Egg mass, Hexavalent chromium

## Abstract

***Leucobacter chironomi*** strain MM2LB^T^ (Halpern et al., Int J Syst Evol Microbiol 59:665-70 2009) is a Gram-positive, rod shaped, non-motile, aerobic, chemoorganotroph bacterium. *L. chironomi* belongs to the family *Microbacteriaceae*, a family within the class *Actinobacteria*. Strain MM2LB^T^ was isolated from a chironomid (*Diptera; Chironomidae*) egg mass that was sampled from a waste stabilization pond in northern Israel. In a phylogenetic tree based on 16S rRNA gene sequences, strain MM2LB^T^ formed a distinct branch within the radiation encompassing the genus *Leucobacter*. Here we describe the features of this organism, together with the complete genome sequence and annotation. The DNA GC content is 69.90%. The chromosome length is 2,964,712 bp. It encodes 2,690 proteins and 61 RNA genes. *L. chironomi* genome is part of the Genomic Encyclopedia of Type Strains, Phase I: the one thousand microbial genomes (KMG) project.

## Introduction

Strain MM2LB^T^ (=DSM 19883^T^ = JCM 17022^T^ = LMG 24399^T^), is the type strain of the species *Leucobacter chironomi *[1]. The genus *Leucobacter * was formed by Takeuchi et al. [2] and currently includes 15 species. Members of this genus were found in a verity of environments including air [2],[3], nematodes [4],[5], sediments with chromium contamination [6], cow dung [7], compost [8], fermented seafood [9], phyllosphere [10] and chironomids (*Diptera*) [1]. 

**Table 1:** **Classification and general features of*Leucobacter chironomi*** strain MM2LB^T^**according to the MIGS recommendations**[42]**], published by the Genome Standards Consortium**[43]**and the Names for Life database**[44]

**MIGS ID**	**Property**	**Term**	**Evidence code**^**a**^
	Current classification	Domain *Bacteria*	TAS [44],[45]
		Phylum *Actinobacteria*	TAS [46]
		Class *Actinobacteria*	TAS [47]
		Order *Actinomycetales*	TAS [47]-[50]
		Family *Microbacteriaceae*	TAS [47],[48],[51],[52]
		Genus *Leucobacter*	TAS [2]
		Species *Leucobacter chironomi*	TAS [1]
		Type strain MM2LB^T^	TAS [1]
	Gram stain	positive	TAS [1]
	Cell shape	rod	TAS [1]
	Motility	Non-motile	TAS [1]
	Sporulation	Non-sporulating	IDS
	Temperature range	17-37°C	TAS [1]
	Optimum Temperature	30°C	TAS [1]
	pH range	4.0-9.5	TAS [1]
	Optimum pH	6.0-8.0	TAS [1]
	Carbon source	Putrescine and Glycerol^b^	TAS [1]
MIGS-6	Habitat	Aquatic/Insect host	TAS [1]
MIGS-6.3	Salinity	0-7.0% NaCl (w/v)	TAS [1]
MIGS-22	Oxygen requirement	Aerobic	TAS [1]
MIGS-15	Biotic relationship	Commensal (Insect, chironomid)	TAS [1]
MIGS-14	Pathogenicity	None	NAS
MIGS-4	Geographic location	Northern Israel	TAS [1]
MIGS-5	Sample collection	July 2006	TAS [1]
MIGS-4.1	Latitude	32.669167	IDS
MIGS-4.2	Longitude	35.128639	IDS
MIGS-4.4	Altitude	40 m	TAS [1]

^a^Evidence codes - IDA: Inferred from Direct Assay; TAS: Traceable Author Statement (i.e., a direct report exists in the literature); NAS: Non-traceable Author Statement (i.e., not directly observed for the living, isolated sample, but based on a generally accepted property for the species, or anecdotal evidence). Evidence codes are from the Gene Ontology project [53].

^b^The only carbon source that was positive for this strain, out of all carbon sources that were tested (strain MM2LB^T^ does not use carbohydrates, not even glucose) [1].

**Table 2:** Genome sequencing project information

**MIGS ID**	**Property**	**Term**
MIGS 31.1	Sequencing quality	Level 2: High-Quality Draft
MIGS-28	Libraries used	Illumina Std. shotgun library
MIGS 29	Sequencing method	Illumina HiSeq 2000
MIGS 31.2	Fold coverage	122.1X
MIGS 30	Assemblers	Velvet (v. 1.1.04), ALLPATHS –LG (v. r42328)
MIGS 32	Gene calling method	Prodigal 2.5
	Locus Tag	H629
	Genbank ID	ATXU00000000
	Genbank Date of Release	12-DEC-2013
	GOLD ID	Gp0013907
	BIOPROJECT	PRJNA188922
MIGS-13	Source Material Identifier	DSM 19883^T^
	Project relevance	GEBA-KMG, Tree of: Life

**Table 3:** Genome statistics

**Attribute**	**Value**	**% of total**
Genome size (bp)	2,964,712	100.00
DNA coding (bp)	2,686,984	90.60
DNA G + C (bp)	2,072,411	69.90
DNA scaffolds	27	100.00
Total genes	2,751	100.00
Protein coding genes	2,690	97.78
RNA genes	61	2.22
Pseudo genes	0	0
Genes in internal clusters	2,248	81.7
Genes with function prediction	2,188	79.53
Genes assigned to COGs	1,842	66.96
Genes with Pfam domains	2,249	81.75
Genes with signal peptides	158	5.74
Genes with transmembrane helices	755	27.44
CRISPR repeats	0	0

**Table 4:** Number of genes associated with the general COG functional categories

**Code**	**Value**	**% age**	**Description**
J	161	8.39	Translation, ribosomal structure and biogenesis
A	1	0.05	RNA processing and modification
K	173	8.39	Transcription
L	108	5.24	Replication, recombination and repair
B	1	0.05	Chromatin structure and dynamics
D	19	0.92	Cell cycle control, cell division, chromosome partitioning
V	34	1.65	Defense mechanisms
T	77	3.74	Signal transduction mechanisms
M	90	4.37	Cell wall/membrane biogenesis
N	0	0	Cell motility
U	23	1.12	Intracellular trafficking, secretion and vesicular transport
O	60	2.91	Posttranslational modification, protein turnover, chaperones
C	109	5.29	Energy production and conversion
G	111	5.39	Carbohydrate transport and metabolism
E	285	13.83	Amino acid transport and metabolism
F	70	3.40	Nucleotide transport and metabolism
H	88	4.27	Coenzyme transport and metabolism
I	83	4.03	Lipid transport and metabolism
P	142	6.89	Inorganic ion transport and metabolism
Q	52	2.52	Secondary metabolites biosynthesis, transport and catabolism
R	240	11.64	General function prediction only
S	144	6.99	Function unknown
-	909	33.04	Not in COGs

**Figure 1:**
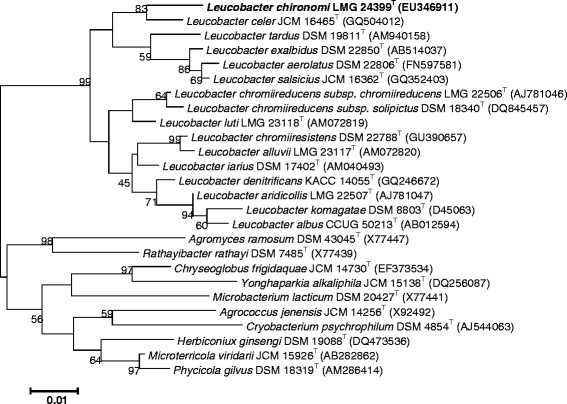
Phylogenetic tree highlighting the position of *Leucobacter chironomi* relative to the type strains of the other species within the genus *Leucobacter.* The sequence alignments were performed by using the CLUSTAL W program and the tree was generated using the maximum likelihood method in MEGA 5 software [41]. Bootstrap values (from 1,000 replicates) greater than 50% are shown at the branch points. The bar indicates a 1% sequence divergence.

**Figure 2:**
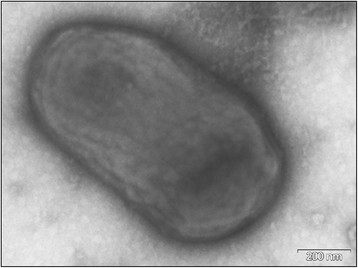
Electron micrograph of negatively stained cells of strain MM2LB^T^. Cells are nonflagellated rods. Bar, 200 nm.

*L. chironomi* MM2LB^T^, was isolated from an insect egg mass (*Chironomus* sp*.*) that was sampled from a waste stabilization pond in northern Israel [1]. Chironomids (*Insecta*; *Diptera*; *Chironomidae*; *Chironomus* sp*.*), also known as the non biting midges, are aquatic insects. They undergo a complete metamorphosis of four life stages; egg, larva, pupa and adult that emerges into the air. The eggs are deposited by the adult female at the water’s edge in egg masses which contain hundreds of eggs [11]. Chironomid egg masses were found as natural reservoirs of *Vibrio cholerae * and *Aeromonas* species [11]-[17]. Strain MM2LB^T^ was isolated in the course of a study that explored the endogenous bacterial communities in chironomid egg masses [1]. Using 454-pyrosequencing technique, Senderovich & Halpern [18], showed that the prevalence of *Leucobacter * in chironomid egg masses and larval endogenous bacterial communities is 0.1% and 0.2%, respectively.

Here we describe a summary classification and a set of the features of *L. chironomi *, together with the genome sequence description and annotation.

## Organism Information

### Classification and features

A taxonomic study using a polyphasic approach placed *L. chironomi * strain MM2LB^T^ in the genus *Leucobacter* within the family *Microbacteriaceae * (order; *Actinomyecetales,* class; *Actinobacteria **,* phylum; *Actinobacteria *) (Figure 1). The family *Microbacteriaceae* comprises more than 40 genera and a large variety of species and phenotypes.

*L. chironomi* strain MM2LB^T^ is a Gram-positive, aerobic, chemo-organotrophic, non-motile single cell rod (Figure 2). After 48 h incubation on LB agar at 30°C, colonies are opaque, circular, with entire margins and yellow-coloured [1]. Growth is observed at 17–37°C (optimum 30°C), with 0–7% (w/v) NaCl (optimum 0–1.0% NaCl) and at pH 4.0–9.5 (optimum pH 6.0–8.0) (Table 1). Oxidase reaction is negative; catalase reaction is weakly positive. Strain MM2LB^T^ produces acetoin and reduces nitrate to nitrogen; H_2_S and indole are not produced; urea and gelatin are not hydrolyzed; citrate is not utilized; β-galactosidase, arginine dihydrolase, lysine decarboxylase, ornithine decarboxylase and tryptophan deaminase activities are absent; Putrescine and glycerol are utilized [1].

#### Chemotaxonomic data

The dominant cellular fatty acids are anteiso-C_15:0_, anteiso-C_17:0_ and iso-C_16:0_. Cell-wall amino acids are alanine, glycine, threonine, DAB, γ-aminobutyric acid and glutamic acid. Strain MM2LB^T^ has a B-type crosslinked peptidoglycan. The major menaquinone is MK-11; MK-10 and MK-12 occur in minor amounts [1]. Strain MM2LB^T^ is able to grow in the presence of up to 18.0 mM Cr(VI) [1].

## Genome sequencing information

### Genome project history

*L. chironomi* MM2LB^T^, was selected for sequencing due to its phylogenetic position [19]-[21], and is part of Genomic Encyclopedia of Type Strains, Phase I: the one thousand microbial genomes (KMG) study [22] which aims not only to increase the sequencing coverage of key reference microbial genomes [23] but also to generate a large genomic basis for the discovery of genes encoding novel enzymes [24]. The sequencing project is accessible in the Genomes OnLine Database [25] and the genome sequence is deposited in GenBank. Sequencing, finishing and annotation were accomplished by the DOE Joint Genome Institute (JGI) [26] using state of the art genome sequencing technology [27]. The project information is summarized in Table 2.

### Growth conditions and genomic DNA preparation

*L. chironomi* MM2LB^T^, DSM 19883, was grown in Trypticase Soy Yeast Extract medium (DSMZ medium 92) at 28°C [28]. DNA was isolated from 0.5-1.0 g of cell paste using Masterpure DNA purification kit (Epicentre MGP04100) following the standard protocol as recommended by the manufacturer with additional 7.5 units of each of the following enzymes achromopeptidase, lysostaphin, mutanolysin and 2100 units of lysozyme, incubated for one hour at 37°C, followed by addition of 1 μl proteinase K and incubation for 20 min at 70°C for cell lysis. DNA is available through the DNA Bank Network [29].

### Genome sequencing and assembly

The draft genome of *L. chironomi * DSM 19883^T^ was generated at the DOE Joint genome Institute (JGI) using the Illumina technology [30]. An Illumina standard shotgun library was constructed and sequenced using the Illumina HiSeq 2000 platform which generated 13,901,154 reads totaling 2,085.2 Mb. All general aspects of library construction and sequencing performed at the JGI can be found at the Institute web site [25]. All raw Illumina sequence data was passed through DUK, a filtering program developed at JGI, which removes known Illumina sequencing and library preparation artifacts (Mingkun L, et al., unpublished, 2011). Following steps were then performed for assembly: (1) filtered Illumina reads were assembled using Velvet [31], (2) 1–3 kb simulated paired end reads were created from Velvet contigs using wgsim [32], (3) Illumina reads were assembled with simulated read pairs using Allpaths–LG [33]. Parameters for assembly steps were: (1) Velvet (velveth: 63 –shortPaired and velvetg: −very clean yes –exportFiltered yes –min contig lgth 500 –scaffolding no –cov cutoff 10) (2) wgsim (−e 0 –1 100 –2 100 –r 0 –R 0 –X 0) (3) Allpaths–LG (PrepareAllpathsInputs: PHRED 64 = 1 PLOIDY = 1 FRAG COVERAGE = 125 JUMP COVERAGE = 25 LONG JUMP COV = 50, RunAllpathsLG: THREADS = 8 RUN = std shredpairs TARGETS = standard VAPI WARN ONLY = True OVERWRITE = True). The final draft assembly contained 27 contigs in 27 scaffolds and is based on 361.8 Mb of Illumina data, which provides an average 122.1X coverage of the genome.

### Genome annotation

Genes were detected using the Prodigal software [34] at the DOE-JGI Genome Annotation pipeline [35],[36]. The CDSs predicted were translated and searched against the Integrated Microbial Genomes (IMG) non-redundant database, UniProt, TIGRFam, Pfam, PRIAM, KEGG, COG, and InterPro databases. Additional gene prediction and functional annotation analysis was carried out in the Integrated Microbial Genomes (IMG-ER) platform [37].

### Genome properties

The assembly of the draft genome sequence consists of 27 scaffolds amounting to 2,964,712 bp, and the G + C content is 69.9% (Table 3). Of the 2,751 genes predicted, 2,690 were protein-coding genes, and 61 RNAs. The majority of the protein-coding genes (79.5%) were assigned a putative function while the remaining ones were annotated as hypothetical proteins. The distribution of genes into COGs functional categories is presented in Table 4.

### Insights from the genome sequence

Senderovich and Halpern [18],[38], demonstrated that endogenous bacteria in chironomids have a role in protecting their insect host from toxic metals. *L. chironomi * strain MM2LB^T^, which was isolated from a chironomid egg mass was found to tolerate up to 18 mM Cr(VI) [1]. Other *Leucobacter * species like *L. alluvii **,**L. aridicollis **,**L. chromiireducens **,**L. chromiiresistens **,**L. komagatae **,**L. luti **and L. salisicius*, have also been found to be resistant to hexavalent chromium [1],[2],[5],[39],[40]. A chromate membrane transport protein A (ChrA) was detected in the genome of the chromate-resistant bacterium, *L. salsicius * M1-8^T^[40]. However, this gene or other genes with chromium reduction predicted functions were not identified in *L. chironomi * MM2LB^T^ genome. Nevertheless, three genes for ABC-type metal ion transport system (permease, ATPase and periplasmic components), were detected in the genome of strain MM2LB^T^. These genes may have a role in *L. chironomi * chromium tolerance.

More genes that may indicate the potential of strain MM2LB^T^ to tolerate or detoxify metals, were also detected. Among them are genes for arsenical resistance: arsenical-resistance protein (*arsB*); arsenite efflux pump ACR3 and related permeases. Other genes suggest the potential of *L. chironomi * to survive in the presence of other toxic metals: copper chaperone; copper-(or silver)-translocating P-type ATPase; heavy metal-(Cd/Co/Hg/Pb/Zn)-translocating P-type ATPase and transcriptional regulator (ArsR family) which is involved in stress-response to heavy metal ions.

Three genes encoding drug resistance transporters are found in strain MM2LB^T^ genome: drug resistance transporter Bcr/CflA subfamily; multidrug resistance efflux transporter and drug resistance transporter EmrB/QacA subfamily. Four copies of Beta-lactamase class C and other penicillin binding proteins were also found in three different domains of strain’s MM2LB^T^ genome.

One gene encoding the two component transcriptional regulator LuxR family is present in the genome of strain MM2LB^T^ and demonstrates quorum sensing skills.

Tolerance of up to 7.0% NaCl was described for strain MM2LB^T^[1]. Three genes for ABC-type proline/glycine betaine transport system (ATP binding subunit, permease and periplasmatic components), that seem to be located in the same operon, are present in strain MM2LB^T^ genome. The accumulation of glycine betaine and other solutes offer osmoprotection, thus, this transport system is probably involved in osmoregulation.

Three genes in *L. chironomi * had best hits with genes from Eukaryotes, indicating a possible horizontal transfer of genes from Eukaryotes to *L. chironomi **.* These genes were: Exodeoxyribonuclease VII small subunit and a protein from PAC2 family, both form Anopheles gambiae origin and a hypothetical protein from Drosophila williston origin. *Anopheles* and *Drosophila* as well as Chironomids belong to the *Diptera* order. *L. chironomi * was isolated from chironomids. Since chironomid species have not yet been sequenced, the horizontal gene transfer from the Diperan origin to *L. chironomi * may point toward the ancient relationships between this bacterium and its chironomid host.

The genome sequences of three more *Leucobacter * isolates have recently been published; L. chromiiresistens, isolated from a soil sample [40]; *Leucobacter* sp. UCD-THU isolated from a residential toilet [54]; and *Leucobacter salsicius * isolated from Korean salt-fermented seafood [39]. Chromate resistance was reported for some of these species (*L. chironomi *, *L. chromiiresistens* and *L. salsicius *) [1],[39],[40]. The genome analysis of *L. salsicius * detected chromate transport protein A (ChrA) that confers heavy metal tolerance via chromate ion efflux from the cytoplasm [39]. In contrast, this gene is not present in the genome of *L. chironomi * and *L. chromiiresistens **.* However, in both strains, other genes for metals tolerance or ion efflux, are present. Interestingly, we have detected a chromate transporter (Chr) gene in the genome of *Leucobacter * sp. UCD-THU, although no evidence for chromate resistance was reported in vivo for this strain [54]. Another interesting feature is the differences in the horizontal gene transfer found in all four *Leucobacter* species genomes. While no horizontal gene transfer from Eukaryotes was detected for *Leucobacter * sp. UCD-THU, we detected horizontal gene transfer from fungi belonging to the phyla *Basidiomycota* and *Ascomycota* in *L. salsicius * and *L. chromiiresistens* genomes, respectively. For *L. chromiiresistens *, which was isolated from seafood, genes transfer from the phylum *Chordata* was also found. Horizontal gene transfer from insects was detected for *L. chironomi * in the current study, confirming the fact that chironomid insects are *L. chironomi * hosts.

## Conclusions

In the current study, we characterized the genome of *L. chironomi * strain MM2LB^T^ that was isolated from a chironomid egg mass [1]. Recently, we have demonstrated that endogenous bacteria in chironomids have a role in protecting their insect host from toxic metals [18],[38]. Genes indicating the potential role of strain *L. chironomi * to tolerate or detoxify metals, where detected in its genome, demonstrating that indeed, *L. chironomi * which inhabits chironomids has a part in protecting its host from toxicants. Genes for ABC-type proline/glycine betaine transport system that were found in the genome may explain the salt tolerance properties of *L. chironomi *. Evidence of horizontal transfer of genes from Diperan origin to *L. chironomi *, implies toward an ancient relationships between *L. chironomi * and its chironomid host.

## Abbreviations

KMG: One thousand microbial genomes

GEBA: Genomic encyclopedia of Bacteria and Archaea

MIGS: Minimum information about a genome sequence

DOE JGI: Department of Energy, Joint Genome Institute

TAS: Traceable

NAS: Non-traceable

## Competing interests

The authors declare that they have no competing interests.

## Authors’ contributions

MH and RP isolated and characterized strain MM2LB^T^; SL, MH, HPK and NCK drafted the manuscript. AL, AC, TBKR, MH, AP, NNI, VMM and TW sequenced, assembled and annotated the genome. All authors read and approved the final manuscript.
